# Impact of histone post-translational modification inhibitors on lifespan, reproduction, and stress response in the rotifer *Brachionus manjavacas*

**DOI:** 10.1371/journal.pone.0324769

**Published:** 2025-10-29

**Authors:** Nelia Luviano Aparicio, Meghan Dryburgh, Colleen M. McMaken, Alyssa Liguori, Kristin E. Gribble

**Affiliations:** Josephine Bay Paul Center for Comparative Molecular Biology and Evolution, Marine Biological Laboratory, Woods Hole, Massachusetts, United States of America; BMSCE: BMS College of Engineering, INDIA

## Abstract

Epigenetic modifications, including histone post-translational modifications, are central drivers of age-associated structural and functional changes in the genome, influencing gene expression and leading to changes in cellular resilience. Epigenetic modifications are thus a target for therapies to prevent or treat age-related decline in health and lifespan. In this study, we measured the effects of inhibiting histone deacetylases (HDACs) and the histone methyltransferase, SETDB1, on lifespan, reproduction, and stress response in the rotifer *Brachionus manjavacas*, a model organism for aging studies. Rotifers were exposed to three pharmaceutical compounds, the HDAC inhibitors β-hydroxybutyrate and sodium butyrate and the SETDB1 inhibitor mithramycin A. Changes in global histone modification levels, lifespan, reproduction, and heat stress resistance were quantified. Global histone acetylation levels increased with β-hydroxybutyrate and sodium butyrate treatments. Histone 3 lysine 9 trimethylation (H3K9me3) levels were reduced by treatment with mithramycin A. β-hydroxybutyrate significantly extended lifespan without modifying heat stress resistance. In contrast, mithramycin A increased both lifespan and heat stress tolerance. Sodium butyrate specifically improved heat stress resistance without affecting lifespan. Importantly, none of the three treatments had a significant impact on lifetime reproduction. These findings provide insights into the role of histone modifications in aging and suggest potential interventions targeting epigenetic marks to promote longevity and resilience.

## Introduction

Epigenetic mechanisms have emerged as key contributors to age-related changes in genome structure and function [1,2]. Among the main types of epigenetic regulation, DNA methylation, histone modifications, and non-coding RNAs modify the architecture and accessibility of DNA and ultimately regulate gene expression [3–5]. In this study, we explored manipulation of epigenetic histone modifications as a means to increase lifespan and extend healthspan.

Post-translational modifications (PTMs) of histones, including acetylation, methylation, phosphorylation, ubiquitylation, and sumoylation, direct chromatin organization and gene expression. Specific enzymes catalyze the addition or removal of these chemical groups from lysine residues on histone tails, resulting in the formation of either condensed heterochromatin or accessible euchromatin. This dynamic restructuring of chromatin determines the accessibility of genomic regions to transcription factors, thereby controlling gene expression. Histone modifications thus orchestrate essential physiological processes, from cell differentiation to organ development, and patterns of histone PTMs evolve throughout an organism’s lifespan. Histone acetylation and methylation are the most extensively studied modifications in the context of aging [6].

Histone deacetylases (HDACs) remove acetyl groups from lysine residues on histones, leading to chromatin condensation and reduced transcriptional activity [7]. Notably, different HDACs have distinct effects on lifespan. For example, deletion of the HDAC *SIR2* shortens the lifespan of *Saccharomyces cerevisiae*, while its overexpression extends lifespan [8]. In contrast, knocking out the HDAC *RPD3* significantly increases yeast lifespan [9]. These contrasting effects illustrate the complexity of HDAC-mediated regulation of aging and emphasize the need to understand how specific HDACs influence longevity. Since aging is often associated with the downregulation or dysregulation of biosynthetic and metabolic pathways, treatment with HDAC inhibitors might delay age-related decline by restoring expression of critical genes. Additionally, inhibiting HDACs may alter expression of genes involved in inflammation and stress responses, pathways closely linked to aging and longevity [10,11]. These findings suggest that HDAC inhibitors hold promise as therapeutic tools for modulating aging processes.

The histone methyltransferase SETDB1 (SET domain bifurcated histone lysine methyltransferase 1, also known as ESET or KMT1E) catalyzes the deposition of di- and tri-methyl marks on H3K9 (H3K9me2 and H3K9me3), which are linked to transcriptional repression [12]. Functional analysis and knockout studies have revealed that SETDB1 is necessary for establishing the methylation marks required for proper oocyte maturation and that *Setdb1* gene expression in mouse ovaries decreases with age [13]. In a mouse strain carrying a triple knockout of three methyltransferases responsible for H3K9me3 deposition, the loss of H3K9me3 in adulthood results in premature aging [14]. A comparable phenomenon was observed in young *Drosophila melanogaster*, in which H3K9me3 is normally concentrated in specific chromosomal regions. As flies age, this site-specific enrichment decreases, leading to similar levels of H3K9me3 across different chromosome regions [15]. These findings suggest that SETDB1 is involved in age-associated alterations in global H3K9 methylation patterns.

Given that histone acetylation and methylation levels and distribution change with age and are associated with age-related changes in gene expression and functional decline, these epigenetic modifications are potential targets for manipulation to extend healthspan and lifespan. Our prior work has shown that the expression of several HDAC genes—including HDAC1, HDAC3, HDAC4, HDAC8—as well as SETDB1, is increased in late life. These findings led us to hypothesize that the activity of these enzymes could be associated with late-life decline, and that lowering their activity could have health and longevity benefits [16]. Pharmaceutical inhibitors of histone modifying enzymes, many of which have been used in treatment of cancer or other diseases, might be used as aging therapies, but relatively few have been tested in this capacity.

HDAC inhibitors prevent the removal of acetyl groups from histones, which can enhance gene expression and thereby possibly extend lifespan. In this work, we tested two HDAC inhibitors, β-hydroxybutyrate and sodium butyrate, that have been shown to increase lifespan in *Caenorhabditis elegans* [17] and *D. melanogaster* [18], respectively. β-hydroxybutyrate is an endogenous ketone molecule that acts as a natural inhibitor of class I HDACs; it serves both as an energy source and as a signaling molecule that influences gene expression, inflammation, and stress resistance through epigenetic modifications [19]. Sodium butyrate is a short-chain fatty acid that selectively inhibits class I and II HDACs, particularly targeting HDACs 1, 2, and 3. Its inhibitory effect is achieved by binding to the active site of HDAC enzymes, blocking their function and enhancing histone acetylation, which in turn promotes the transcription of genes involved in cell cycle regulation, apoptosis, and differentiation [20].

Because of the association of high SETDB1 expression with reproductive aging in mice [13], we additionally explored the lifespan and health effects of the SETDB1 inhibitor, mithramycin A. Mithramycin A is an antibiotic and anticancer compound known primarily for its ability to bind GC-rich DNA sequences, thereby inhibiting the transcription factor Sp1 (specificity protein 1) and affecting gene expression [21]. Recently, it has also been studied for its potential to inhibit histone methyltransferase SETDB1 [22]. SETDB1 is crucial for gene silencing and for maintaining heterochromatin structure, particularly in regions associated with oncogenesis and other diseases [23]. By blocking SETDB1 activity, mithramycin A reduces H3K9me3 levels, leading to increased chromatin accessibility and the reactivation of silenced genes [24].

To assess whether histone modification inhibitors could be used as therapies to increase longevity and improve health, we quantified age-specific gene expression of histone deacetylases and SETDB1 and measured the effects of β-hydroxybutyrate, sodium butyrate, and mithramycin A on histone modification levels, lifespan, reproduction, and stress resistance. This work was conducted using the rotifer *Brachionus manjavacas,* a microscopic, aquatic invertebrate with a short life cycle, rapid generation time, easy laboratory culture, and high genetic homology with humans, making it a useful model system in which to study aging [25]. Our findings will provide insights into how modulation of histone modifications can influence lifespan, reproduction, and stress resistance, paving the way for novel therapeutic interventions to increase longevity and healthspan.

## Materials and methods

### Rotifer and algae culture

*Brachionus manjavacas* “L5 strain” was fed the chlorophyte *Tetraselmis suecica*, which was cultivated in 2 L flasks containing bubbled f/2 medium [26], excluding silica, prepared with 15 ppt Instant Ocean Sea Salt (Instant Ocean, Blacksburg, VA) made with filtered deionized water. Both the rotifer and algae cultures were maintained at 21°C under a 12:12 h light:dark cycle. Cultures of *T. suecica* were kept in semi-continuous logarithmic growth phase by removing approximately 40–50% of the culture volume and replacing it with f/2 medium every two days throughout the experiments.

### Gene expression analysis

Age-specific gene expression of histone modifiers was collected as part of an RNA-Seq study. Multiple generations of age synchronization were conducted before rotifers were collected. mRNA was extracted from five replicates of pelleted rotifers from each of four age cohorts: one, three, six, and 10-days old (~400 rotifers per pellet for the one-day timepoint and ~200 rotifers per pellet for later ages)*.* RNA was extracted following the TRIzol™ reagent protocol (#15596026, Invitrogen), with residual DNA removed using Turbo DNA-free (#AM1907, Invitrogen), and RNA precipitated with isopropanol.

The quality, quantity, and purity of RNA samples were evaluated using an Agilent 2100 Bioanalyzer (Agilent Technologies), Qubit™ 2.0 Fluorometer (Invitrogen), and NanoDrop™ 2000 spectrophotometer (Thermo Fisher Scientific). cDNA libraries were constructed with poly-A enrichment from the RNA samples using the NEB Next Ultra II RNA kit for Illumina (#E7770, New England Biolabs). Sequencing was performed on an Illumina NovaSeq platform by Novogene Bioinformatics Technology, generating 150 bp paired-end reads.

The RNA-Seq data were processed through a bioinformatics pipeline. Raw reads were trimmed using FASTX [27] and Cutadapt [28] to remove adapter sequences and low-quality bases. The cleaned reads were mapped to a reference genome using Bowtie2 [29,30]. The transcriptome was assembled with a genome-guided approach using Trinity [31]. The resulting transcripts were annotated using blastx [32] against the NCBI Nucleotide (nt) database. Sequences that yielded significant hits to non-metazoan organisms were excluded from the dataset (e-value < 1e-6). Transcript abundance was quantified using Salmon [33], which accounted for transcript length and sequencing depth to estimate the number of reads corresponding to each gene and gene isoform. Normalization of gene counts was done using the median of ratios method in DESeq2 [34].

### Survival and reproduction assays

Lifespan and reproduction were measured as in previous studies [35,36]. To synchronize age and to control for maternal and grandmaternal ages of the experimental animals, two generations of maternal age synchronization were conducted prior to beginning life table experiments. Amictic eggs were removed from females by vortexing, isolated by micropipette, and allowed to hatch and mature for five days in *ad libitum* food conditions, at which time eggs were again collected from mature females. After repeating this for two generations, eggs were collected and hatched overnight to initiate the experimental cohort of same-aged individuals.

To initiate and track each cohort, neonates were deposited individually into 0.5 mL of *T. suecica* at a concentration of 6 × 10^5^ cells mL^−1^ in 15 ppt Instant Ocean and test drugs (as specified below) in 24-well plates (n = 72 per treatment). Individuals were observed every 24 h on a Zeiss Stemi 508 microscope, and survival, reproductive status (carrying or not carrying eggs), and the number of new offspring were quantified. The original female was then transferred to a well with new *T. suecica* and drug in 15 ppt Instant Ocean. Death was characterized by the cessation of swimming and of movement in the cilia, muscles, and mastax (jaw). Daily scoring and transfers were conducted until all individuals had died.

### Drug testing

β-hydroxybutyrate (#H6501, Sigma-Aldrich), sodium butyrate (#303410, Sigma-Aldrich) and mithramycin A (#11434, Cayman Chemical) were used to treat rotifers. Prior to conducting survivorship and reproduction assays, we tested 100 µM, 500 µM and 1 mM for β-hydroxybutyrate; 50 µM, 100 µM and 500 µM for sodium butyrate; and 100 nM, 500 nM and 1 µM for mithramycin A in a 3-day trial to determine optimal concentrations. Based on survivorship results, daily treatments for full experiments consisted of 1 mM β-hydroxybutyrate, 500 µM sodium butyrate, or 500 nM mithramycin A, which were added to experimental wells containing *T. suecica* in 15 ppt Instant Ocean (n = 72 per treatment). Instant Ocean and 0.01% DMSO were used as controls.

### Heat stress assay

To assess the impact of histone modification inhibitors on heat stress resistance, neonate rotifers were exposed to 1 mM β-hydroxybutyrate, 500 µM sodium butyrate, 500 nM mithramycin A, or 0.1% DMSO (control) for two days. Following treatment, 5 rotifers were placed in each of the 8 central wells of a 24-well plate (n = 40), with each treatment allocated to a separate plate. The plates were sealed with lab tape and floated in a 42 °C water bath for one hour to induce heat stress. After heat exposure, the plates were transferred to 21 °C. For each treatment, the number of live and dead rotifers was recorded at 24- and 48-hours post-heat exposure.

### Quantification of histone modifications

To assess whether histone modification inhibitors altered histone post-translational modifications (PTM) levels in *B. manjavacas*, three replicate 150 mL batch cultures were treated for three days with 500 µM β-hydroxybutyrate, 1 mM sodium butyrate, or 500 nM mithramycin A, with 0.01% water or DMSO as controls. After three days, rotifers were filtered onto a 100 µm sieve, transferred to 15 ppt Instant Ocean in a 6-well plate, and subjected to overnight starvation at 21°C to clear their guts of algae prior to histone extraction*.*

### Histone extractions

Histone extractions from *B. manjavacas* were performed using a hybrid protocol that combines elements of the acid extraction method [37] with additional steps for subcellular fractionation, aimed at removing non-nuclear proteins more effectively. All steps were performed at 4 °C. Rotifers were pelleted via centrifugation at 10,000 rpm for five minutes. The rotifer pellets were resuspended in 500 µL of fresh, ice-cold hypotonic lysis buffer (HLB: 150 mM MgCl₂, 100 mM KCl, 1 M Tris-HCl [pH 8.0], 1M DTT, protease inhibitors, and nuclease-free water) and homogenized on ice using a Dounce homogenizer. The resulting homogenate was centrifuged at 2,500 rpm for 10 minutes in a QIAshredder homogenizer (#79656, Qiagen), and the pellets were resuspended in the filtrate and incubated in 500 µL of fresh HLB for 30 minutes. Following centrifugation at 10,000 rpm for 10 minutes, the pellets were resuspended in 1 mL of fresh, ice-cold lysis buffer B (LBB: 750 mM NaCl, 1 M Tris-HCl [pH 8.0], 1% Ipegal, 1 M hexylene glycol, protease inhibitors, and nuclease-free water) and rotated at 4 °C for 30 minutes to further isolate nuclear components. After centrifugation at 8,500 rpm for 10 minutes, the pellets were resuspended in 400 µL of 0.4 N H₂SO₄, wrapped in parafilm, and rotated overnight to extract histones.

The samples were centrifuged at 13,000 rpm for 10 minutes to pellet debris, and the histone-containing supernatant was transferred to a new sterile tube. To precipitate the histones, 66 µL of trichloroacetic acid was added to the supernatant, followed by a 30-minute incubation on ice. The samples were centrifuged at 13,000 rpm for 10 minutes, and the resulting pellets were washed twice with 50 µL of ice-cold acetone. After the final wash, the acetone was removed, and the pellet was air-dried for 30 minutes. The dried histone pellets were resuspended in 50 µL of nuclease-free water and incubated in ice for 30 minutes. The histone concentration was determined using a Bradford protein assay [38] to ensure adequate protein yield for downstream analyses.

### Western blot and dot blot analyses

Specificity and sensitivity of all antibodies targeting histone modifications and Histone 3 were validated against rotifer histone protein extracts by Western blot (S1 Fig in S1 File; S3 File). Histone 3 and histone modifications were quantified using dot blot analyses (S2 Fig in S1 File; S3 File). Polyvinylidene fluoride (PVDF) membranes were used for Western blots, and nitrocellulose membranes were used for dot blots. Although both serve similar purposes in protein detection, nitrocellulose was chosen for dot blots due to its higher protein-binding capacity and better performance with direct sample application.

For Western blots, two replicates for each of controls, β-hydroxybutyrate-treated, and mithramycin A-treated samples were separated by SDS-PAGE on replicate gels loaded with the same samples and concentrations. Five micrograms of histone extract were added to all lanes of the gel for all treatments. Gels were transferred overnight onto PVDF membranes using a wet-transfer apparatus at 40 V.

For dot blots, histone extracts from controls (water and DMSO-treated), β-hydroxybutyrate, sodium butyrate, and mithramycin A-treated samples were analyzed. Three technical replicates for each of three biological replicates per treatment or control were spotted onto nitrocellulose membranes, followed by UV crosslinking using a Stratalinker® UV Crosslinker (Autocrosslink setting). All spots were 356 ng of protein. As a positive control, histones from sodium butyrate-treated HeLa cells (#3620, Active Motif) were included to validate the assay’s sensitivity to histone acetylation. An unmodified histone H3 peptide (#ab7228, Abcam) served as a negative control, ensuring specificity for modified histones.

For Western and dot blots, membranes were blocked with TBST (1 × TBS 0.05% Tween 20) and 5% powdered milk for one hour at room temperature under elliptical agitation. Membranes were incubated separately overnight with primary antibody (anti-histone H3,24834, Abcam; anti-histone H3 pan-acetyl, ab300641, Abcam; or anti-histone H3K9me3, #A-4036, Epigentek) at a 1:1000 dilution in blocking solution.

After primary antibody incubation, the membranes were washed for 10 minutes three times with TBST under elliptical agitation and subsequently incubated for one hour at room temperature in a 1:10000 dilution of secondary antibody. HRP-conjugated Goat anti-Rabbit IgG (H + L) secondary antibody (#AS014, ABClonal) was used for the membranes previously incubated with anti-Histone 3 pan-acetyl or anti-H3K9me3. HRP-conjugated Goat anti-Mouse IgG (H + L) (#AS003, ABClonal) was used for the membranes previously incubated with anti-Histone 3. The membranes were washed again three times with TBST and developed using SuperSignal™ West Pico chemiluminescent substrate (#34096, Thermo Fisher Scientific).

Chemiluminescent signal was captured using an Amersham™ Imager 600 with a 60-second exposure time. Dot intensity was quantified using ImageJ software, measuring the integrated density of each dot following the user guide [39].

### Statistical analyses

Statistical analyses were performed in RStudio. The “tidyverse” [40] package was used for data manipulation and visualization, incorporating functions from “ggplot2” [41] for creating graphics and “dplyr”[42] for efficient data wrangling. After testing for normality of the data distributions with the Shapiro-Wilk test and for homogeneity of variances with the Levene’s test for Histone 3 or histone modification levels, unpaired two-sample Student’s t-tests or ANOVA were used to test for differences between treatments and controls.

Kaplan-Meier survivorship curves were generated to estimate the probability of survival over time for each treatment group (n = 72 per group) with the “survival” package [43]. Pairwise comparisons between treatment groups were conducted using the log-rank test (Mantel-Cox) with Benjamini-Hochberg correction for multiple comparisons. Statistical significance was set at *p* < 0.05. Survivorship curves were visualized using the “survminer” package [44].

To assess differences in reproductive output across treatments, normality and homogeneity of variance were tested using Shapiro-Wilk and Bartlett’s tests. Given that the data violated parametric assumptions (*p* < 0.05 for both tests), Kruskal-Wallis tests were used to evaluate differences in reproductive metrics (lifetime reproductive output, total non-viable eggs per individual, and reproductive period) among treatments. When the Kruskal-Wallis test revealed significant differences (*p* < 0.05), post-hoc pairwise comparisons were performed using Wilcoxon rank-sum tests with Bonferroni correction to control for multiple comparisons.

Non-parametric comparisons between groups exposed to heat-stress were performed using the Friedman test, followed by Wilcoxon signed-rank post-hoc tests to compare each treatment against the controls using the “coin” package [45].

## Results

### Gene expression

**Fig 1:**
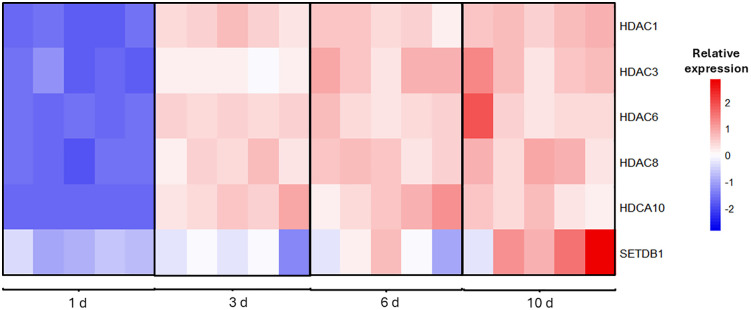
Relative gene expression levels of histone deacetylase enzymes (HDACs) and the methyltransferase SETDB1 enzyme in the rotifer *Brachionus manjavacas* across lifespan (one, three, six, and 10 days old). Columns depict biological replicates, with five replicates per age.

Our transcriptomic study of aging in *Brachionus manjavacas* revealed a progressive increase with age in the expression of HDACs and SETDB1 across four age cohorts (one, three, six, and 10 days old) (Fig 1, S1 Table in S2 File). The expression of HDACs 1, 3, 6, 8, and 10 and SETDB1 was lowest in early life (one day old), increased through mid-life (three – six days old), and reached peak expression at 10 days old.

### Histone modification levels

**Fig 2:**
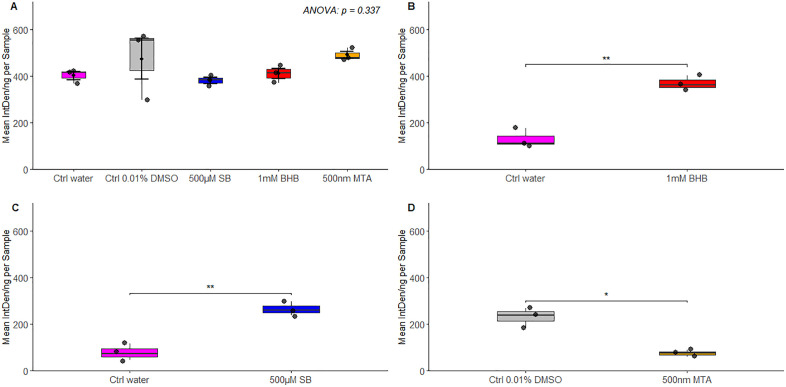
Histone H3 (A), histone acetylation (B, C), and histone methylation (D) levels in *B. manjavacas* measured by dot blot analysis using the antibodies anti-Histone 3, anti-Histone H3 (acetyl K4 + K9 + K14 + K18 + K23 + K27), and anti-Histone H3K9me3 (H3K9 Trimethyl). The black dots represent the mean integrated density of three technical replicates for each of three biological replicates. Boxes represent the interquartile range (IQR) from the 25th to 75th percentile, and error bars denote the standard error of the mean (SEM). Treatments and controls include: Ctrl water = 0.01% water control; Ctrl DMSO = 0.01% DMSO control; BHB = 1 mM β-hydroxybutyrate; SB = 500 µM sodium butyrate; MTA = 500 nM mithramycin A.* indicates p < 0.05 and ** indicates p < 0.01.

Dot blot analyses were conducted to quantify changes in histone modification levels in rotifers following treatment with β-hydroxybutyrate, sodium butyrate, or mithramycin A (Fig 2). To ensure that the drugs tested did not alter total histones, we examined histone H3 levels across all treatments. None of the treatments significantly altered the total histone H3 content in *B. manjavacas* (Fig 2A). Histone acetylation levels increased significantly in response to treatment with the deacetylation inhibitors β-hydroxybutyrate (*t* = 3.38, *df* = 8, *p* = 0.009) and sodium butyrate (*t* = 4.65, *df* = 8, *p* < 0.001), indicating decreased deacetylation activity compared to controls (Fig 2B and 2C). Mithramycin A treatment led to a significant reduction in H3K9me3 levels (*t* = 2.38, *df* = 16, *p* = 0.02) (Fig 2D). These data collectively suggest that the observed changes in epigenetic modifications levels are due specifically to alterations in post-translational modifications and not to changes in histone H3 abundance.

### Lifespan and reproduction effects of histone modification inhibitors

**Fig 3:**
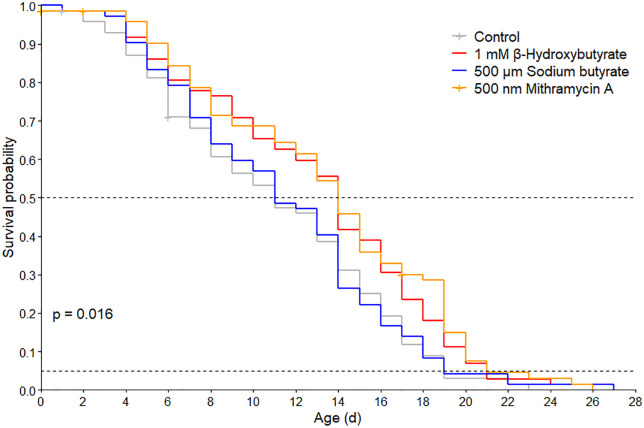
Survivorship of *B. manjavacas* in control group (grey) and under three drug treatments: β-hydroxybutyrate (red), sodium butyrate (blue) and mithramycin A (orange). The *p*-value represents the Mantel Cox test across all four survivorship curves. Dashed lines indicate median (50% survivorship) and maximum (5% survivorship) lifespan.

**Fig 4:**
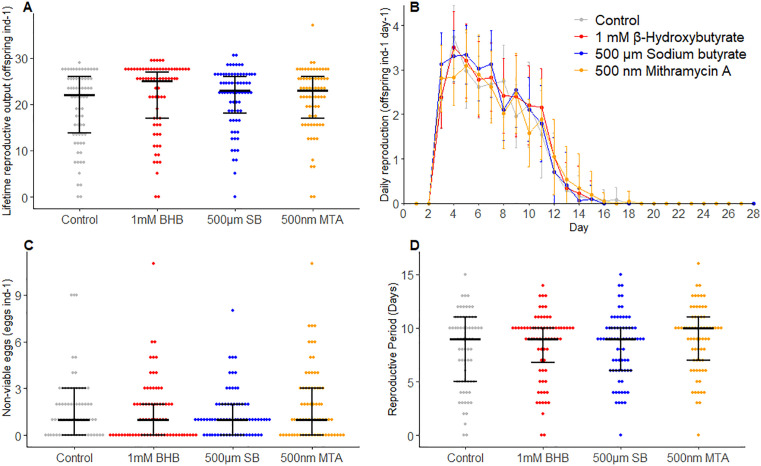
Measures of reproduction in *B. manjavacas* across control and treatment groups: β-hydroxybutyrate (BHB), sodium butyrate (SB), and mithramycin (MTA): (A) Lifetime reproductive output (total offspring per individual), (B) daily reproduction (daily offspring per individual), (C) total non-viable eggs per individual, and (D) reproductive period (in days). The bars show the median values, and the error bars represent the interquartile range from the 25th to 75th percentile.

Two of the three histone modification inhibitors tested significantly extended lifespan in *B. manjavacas.* β-hydroxybutyrate and mithramycin A significantly extended median and maximum lifespan relative to the control, while sodium butyrate treatment resulted in a survivorship trend similar to that of the control group (Fig 3; pairwise log-rank tests, p = 0.030 and p = 0.006, respectively). Median lifespan was 11 days for the control and sodium butyrate-treated groups and 14 days for the β-hydroxybutyrate and mithramycin A treated groups. Similar trends were seen for maximum lifespan (5% survivorship) which was 19 days for the control and sodium butyrate treated groups and increased to 21 days for β-hydroxybutyrate and mithramycin A treated groups (Fig 3, S2 Table in S2 File). Reproductive measures, including lifetime reproductive output, reproductive period, and the number of non-viable eggs, were not significantly affected by any of the drug treatments (Fig 4, S3 Table in S2 File).

### Effect of histone modification inhibitors on heat stress resistance

**Fig 5:**
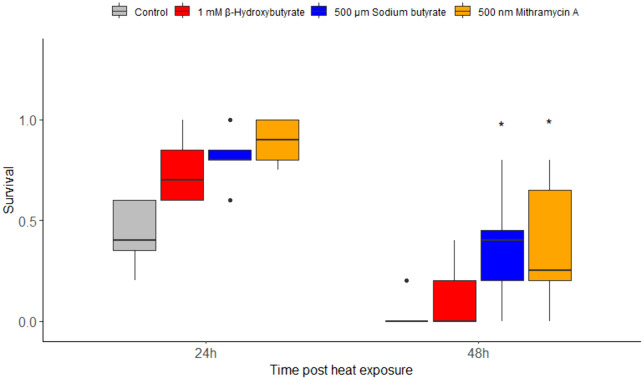
Heat stress resistance of *B. manjavacas* at 24- and 48-hours after heat exposure. The horizontal bars in the plots show the median values, the boxes represent the interquartile range (IQR) from the 25th to the 75th percentile, and error bars denote range. Statistical significance between control and treated groups is denoted by asterisks, where * indicates p < 0.05.

Histone modification inhibitor treatments significantly enhanced rotifer survival relative to that of the control at both 24- and 48-hours following heat exposure (Friedman test, maxT = 4.64, p < 0.0001). Specifically, sodium butyrate and mithramycin A markedly increased the rotifers’ ability to withstand elevated temperatures at both time points, with survival rates significantly higher than the control group (p < 0.005 for each). In contrast, β-hydroxybutyrate exhibited a more modest protective effect, resulting in survival improvements that approached but did not achieve statistical significance (p = 0.08) (Fig 5, S4 Table in S2 File).

## Discussion

Epigenetic modifications are increasingly being shown to be important as controls on health and lifespan rather than simply being marks correlated with biological age [46,47]. The reversible nature of epigenetic marks makes them promising targets to modulate aging rate and phenotype. In this study, we investigated the use of histone modification inhibitors as a means to extend lifespan and increase stress resistance.

We found that gene expression of HDACs 1, 3, 5, 6, 8, and 10 and of the histone methyltransferase SETDB1 increase with increasing age in *B. manjavacas*. Our results confirm the findings in prior transcriptomic analysis of aging in *B. manjavacas,* which showed a similar age-related increase in expression of HDACs 1, 3, 6, and 8 [16]. The previous study sampled at different, less specific ages: eggs, neonates (one – three hours old), early reproductive females (36 hours old), mixed-age reproductive females (three – six days old), and post-reproductive females (six – nine days old). HDACs expression increased with age, without major changes in histone acetylases. Histone demethylases were slightly upregulated, including KDM1A, which rose 0.4-fold during reproduction but declined afterward [16]. These age-related increases in HDACs and SETDB1 expression would be expected to result in decreased levels of histone acetylation and in increased histone methylation, leading to a more condensed chromatin structure and reduced gene transcription [48].

Results in rotifers are thus similar to those from some vertebrate species in which age-related changes in the expression of HDACs and histone methyltransferases have been shown to impact chromatin structure and gene regulation. High levels of HDACs are also detected in aged mice and human brain samples. Relative expression of HDACs increases with age in human cerebral white matter and is associated with age-related changes in its structure; a post-mortem study confirmed that HDAC 1 and HDAC 2 paralogs are significantly elevated in white matter tissue from elderly individuals [49]. HDAC 1, 2, 3, 6, and 7 were found to be specifically intense in the hippocampal formation in adult mice, while a significant elevation of HDAC 1, 3, and 7 was found in the hippocampus in aged human brain samples [50]. Conversely, in the short-lived fish *Nothobranchius furzeri*, HDAC 1 expression decreases with age in muscle, liver, and brain tissues [51].

Less information about the age-related expression of HDACs 1–8 is available for invertebrate models. Knockdown experiments targeting HDACs have been conducted in *D. melanogaster* to explore the enzymes’ roles in aging. The knockdown of HDAC 6 in flies led to significant extensions in both lifespan and healthspan, with the most pronounced effects observed when HDAC 6 was silenced specifically in neuronal tissues [52]. These findings suggest that age-associated alterations in histone-modifying enzymes like HDAC 6 are conserved across species and contribute to aging through epigenetic regulation.

The age-associated increases in histone deacetylation and H3K9me3 that accompany changes in expression of HDACs and SETDB1 prompted the central hypothesis of our study: that inhibiting these enzymes could extend lifespan and enhance stress resilience. Thus, we pharmacologically altered histone acetylation and methylation using drugs that inhibit histone modifiers. We found a significant increase in histone acetylation levels upon treatment with the deacetylation inhibitors β-hydroxybutyrate and sodium butyrate, indicating reduced deacetylation activity compared to controls. This increase suggests that β-hydroxybutyrate and sodium butyrate treatments might promote a more open chromatin state conducive to increased gene expression.

Treatment with β-hydroxybutyrate extends the lifespan of *B. manjavacas* compared to untreated controls. These findings in rotifers are consistent with studies in *C. elegans*, where β-hydroxybutyrate extends lifespan in a concentration-dependent manner [17]. β-hydroxybutyrate also increased the mean survival of worms by 22% at elevated temperatures, suggesting enhanced thermotolerance. However, no induction of heat shock protein expression was observed in four heat shock reporter strains (*hsp-6::gfp*, *hsp-60::gfp*, *hsp-4::gfp*, and *hsp-16.2::gfp*), suggesting that while β-hydroxybutyrate improves thermotolerance, it does not activate a generalized heat shock response. In our study, β-hydroxybutyrate slightly improved heat stress resistance in *B. manjavacas.* This effect may be attributed to increased histone acetylation that promotes the expression of stress response genes that help mitigate heat-induced cellular damage [53].

Sodium butyrate did not significantly extend lifespan of *B. manjavacas*. Our results differ from those in *D. melanogaster*, where the impact of sodium butyrate on lifespan varies depending on the timing of administration. When given during senescent stages, sodium butyrate reduces mortality rates and increases longevity in flies, whereas administration exclusively during early life or throughout the entire adult lifespan decreases longevity [54]. These complex, stage-specific effects align with the nuanced roles that histone acetylation plays in aging and stress resistance across species. It is possible that administering sodium butyrate only in late life in rotifers could yield different effects on lifespan. In our study, sodium butyrate had no significant impact on reproductive output in *B. manjavacas*, but it did enhance heat stress resistance. This suggests that its primary benefits may relate more to stress resilience rather than to reproduction or lifespan, likely due to its role in maintaining histone acetylation levels that modulate stress-responsive gene expression [55]. Histone 3 acetylation may thus be an important regulatory mechanism in the adaptive response to thermal stress.

Mithramycin A has been studied for its ability to bind DNA and modulate gene expression, particularly in mice and in cell culture systems in the context of cancer and neurological research [23,56,57]. Given its role as an inhibitor of Sp1 transcription factors [21], mithramycin A has the potential to be used to investigate epigenetic modifications and gene regulation in aging. We tested mithramycin A for new uses in aging using rotifers, because research on mithramycin A in common invertebrate laboratory models (e.g., *C. elegans*) is limited [57].

Mithramycin A treatment led to a significant reduction in H3K9me3 levels in *B. manjavacas.* This decrease in repressive methylation marks may allow reactivation of previously silenced genes. The increase in lifespan upon mithramycin A treatment may be mediated by inhibition of the transcription factor Sp1, which interacts with SETDB1, affecting gene expression patterns relevant to aging and cellular stress responses [21]. Unlike the other treatments, mithramycin A increased the reproductive period slightly. While the enhancement was not statistically significant, the observed trend warrants further investigation to understand the nuanced role of SETDB1 inhibition on reproduction [13]. Furthermore, mithramycin A enhanced heat stress resistance, suggesting that the observed reduction in H3K9me3 regulates genes involved in heat stress adaptation. This observation is consistent with findings in plant species, which indicate that histone methylation, specifically histone 3 lysine 4 dimethylation, is essential for regulating thermotolerance by influencing the transcription of heat stress-related genes [58]. Collectively, these results highlight that alterations in histone modifications are fundamental mechanisms allowing adaptation to thermal stress.

None of the drugs tested decreased lifetime reproductive output or increased the number of non-viable embryos significantly, suggesting that the observed improvements in lifespan and stress resistance were not due to energy or resource trade-offs with fecundity. The results further suggest that the global changes in histone deacetylation and H3K9me3 levels caused by the tested compounds do not negatively affect development in *B. manjavacas.*

The impact of HDAC or methyltransferase inhibition is context- and residue-specific [59]. Blocking these modifiers will have the largest effect at loci in which acetyl or methyl marks turn over rapidly, e.g., H3K9 and H3K27 in promoters. At sites that are constitutively modified or unmodified, however, additional HDAC inhibition is expected to confer little or no change in acetyl or methyl levels [60].

In our study, we observed changes in global modification levels upon drug treatment, suggesting that the histone modification inhibitors tested affected acetylation and methylation levels. While our assays do not provide information about site-specific changes in modifications, the phenotypic benefits conferred by drug treatment suggest that the inhibitors tested affect dynamically regulated genomic regions that determine longevity and stress resistance. Future experiments employing residue-specific antibodies or ChIP-seq will be required to determine which genomic compartments and which histone lysine sites drive the drug-associated lifespan and stress-resistance phenotypes in *B. manjavacas*.

## Conclusions

This study investigated the important role of histone modifications in regulation of lifespan and stress response and demonstrated the potential of HDAC and SETDB1 inhibitors as therapeutic agents to promote longevity and improve health. How the global changes in histone acetylation and H3K27me3 levels induced by the drugs tested here led to beneficial outcomes remains to be determined. We hypothesize that β-hydroxybutyrate, sodium butyrate, and mithramycin A act by upregulating the expression of genes that are normally down-regulated in late age. Those genes that typically maintain high late-life expression presumably are not further upregulated by opening of chromatin and thus would likely not be impacted by drug treatment. Future research should explore the activity of genes affected by these compounds, as well as the effects of additional histone modification inhibitors, to elucidate the molecular mechanisms underpinning their impact on aging.

## Supporting information

S1 File S1 and S2 Figs. Western blot and dot blot images.
(DOCX)

S2 File S1, S2, S3, and S4 Tables. Data files for gene expression, survivorship, reproduction, and heat stress response.
(XLSX)

S3 File Original Western blot and dot blot images. (PDF)
