# Supplementary material for: Impact of histone post-translational modification inhibitors on lifespan, reproduction, and stress response in the rotifer Brachionus manjavacas
Source: PLoS One. 2025 Oct 29;20(10):e0324769. doi: 10.1371/journal.pone.0324769 (PMC12571253; doi:10.1371/journal.pone.0324769)
Supplement: S1 File — Western blot and dot blot images. (DOCX) [file pone.0324769.s001.docx]

**Supplementary file 1**


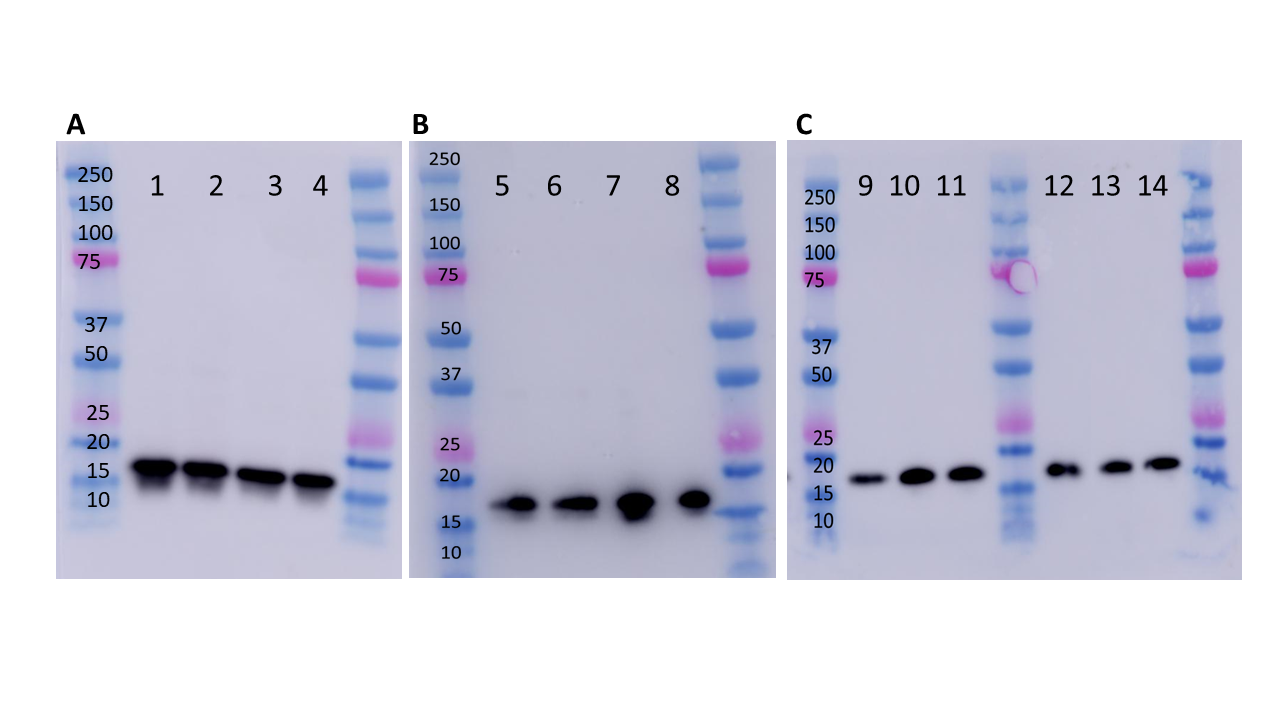


**Figure S1**. *B. manjavacas* histone extracts were analyzed by Western blotting for (A) Histone 3 pan-acetylation, (B) histone 3 K9 trimethyl, and (C) histone 3, to demonstrate specificity of commercial antibodies for *B. manjavacas* proteins. (A) Histone 3 pan-acetylation. Lanes 1-2 are histone extracts from β-hydroxybutyrate-treated culture. Lanes 3-4 are from untreated cultures. (B) Histone 3 K9 trimethyl. Lanes 5-6 are histone extracts from mithramycin A-treated cultures. Lanes 7-8 are from untreated cultures. (C) Western blotting for histone 3: lanes 9-11 are histone extracts from β-hydroxybutyrate-treated cultures, lanes 12-14 are from untreated cultures, and lanes 13-14 are histone extracts from mithramycin A-treated cultures. Two micrograms of total protein were loaded into each gel lane.

**
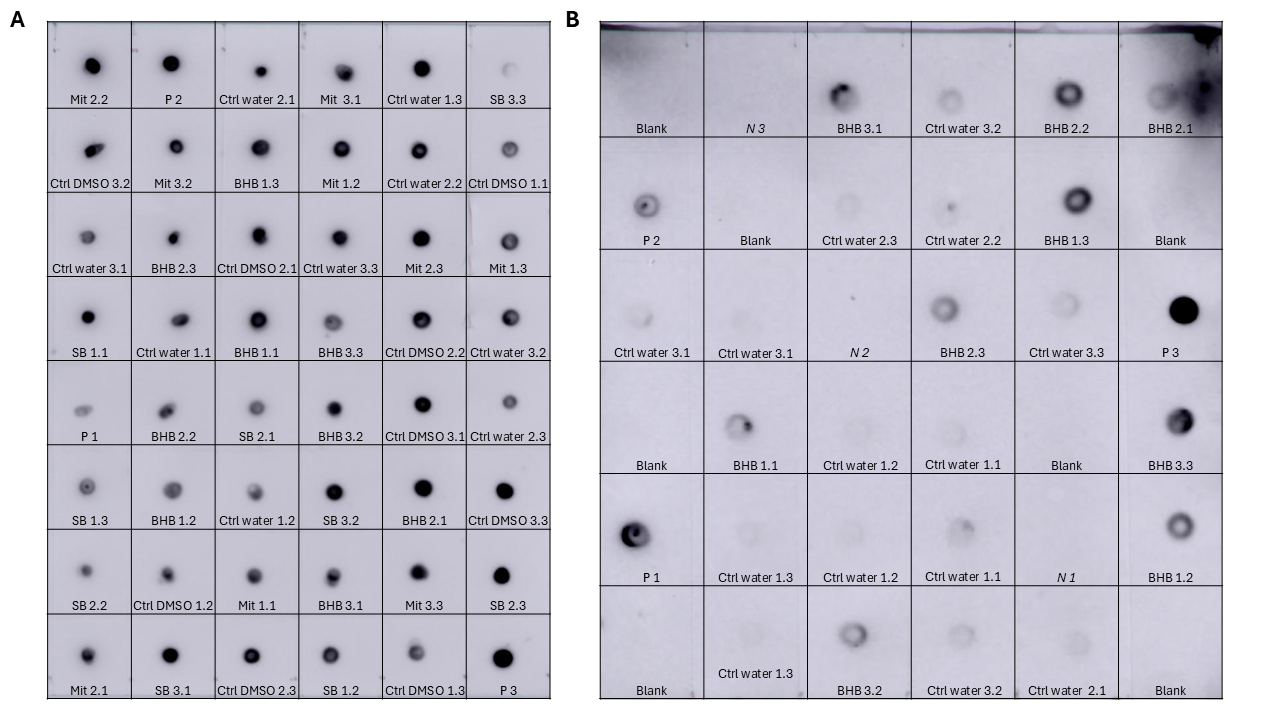
**

**
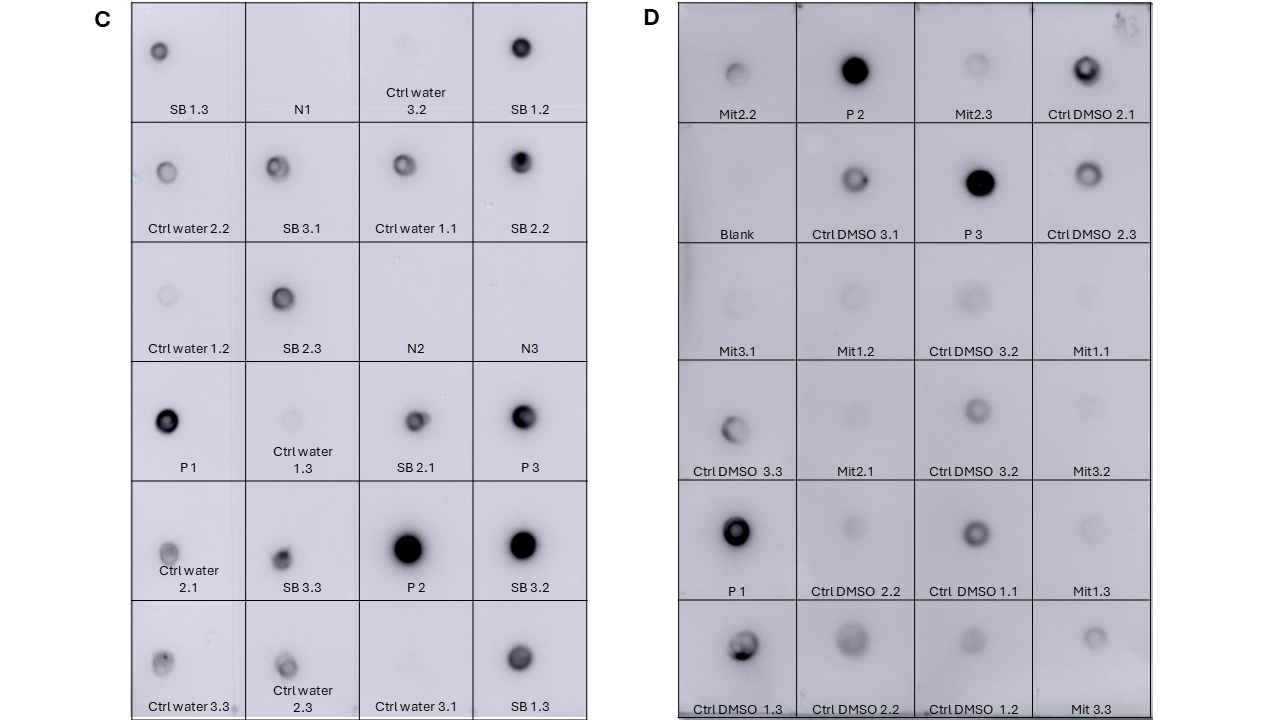
**

**Figure S2.** Dot blot images of (A) histone H3 levels and (B-D) histone modification levels in control and treated samples. (A) Samples from all treatments and controls probed with anti-histone H3 antibody to detect total histone H3 levels. (B) Control (water) and β-hydroxybutyrate-treated samples probed with anti-histone H3 pan-acetyl antibody to detect histone H3 acetylation. (C) Control (water) and sodium butyrate-treated samples probed with anti-histone H3 pan-acetyl antibody to detect histone H3 acetylation. (D) Control (DMSO) and mithramycin A-treated samples probed with anti-H3K9me3 antibody to detect histone H3 lysine 9 trimethylation. Samples were arranged randomly to minimize bias. N denotes the negative control (unmodified histone H3 peptide) and P the positive control (sodium butyrate–treated HeLa cells). Ctrl water = 0.01% water control; Ctrl DMSO = 0.01% DMSO control; BHB = 1 mM β-hydroxybutyrate; SB = 500 µM sodium butyrate; MTA = 500 nM mithramycin A.
