# Supplementary material for: Impact of histone post-translational modification inhibitors on lifespan, reproduction, and stress response in the rotifer Brachionus manjavacas
Source: PLoS One. 2025 Oct 29;20(10):e0324769. doi: 10.1371/journal.pone.0324769 (PMC12571253; doi:10.1371/journal.pone.0324769)
Supplement: S3 File — (PDF) [file pone.0324769.s003.pdf]

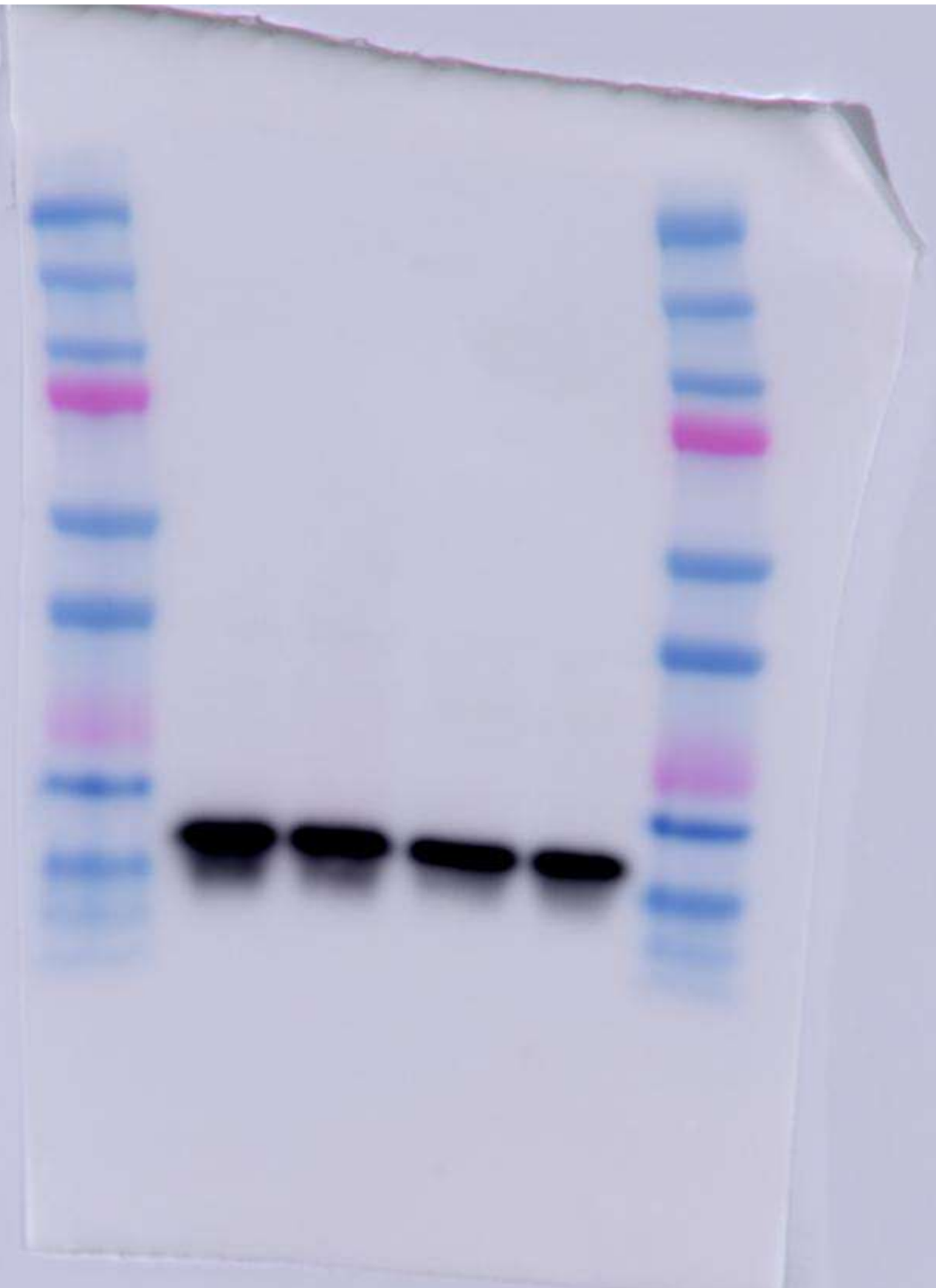

Western blot of Histone 3 pan-acetylation levels measured in histone extracts with the primary antibody: anti-Histone H3 (acetyl K4+K9+K14+K18+K23+K27) ab300641, Abcam. Lane 2 and 3 are β-hydroxybutyrate-treated samples. Lane 4 and 5 are untreated rotifers cultures. Lanes 1 and 6 are the Plus Protein™ Standard used as molecular weight marker.

HRP-conjugated Goat anti-Rabbit IgG (H+L) secondary antibody (#AS014, ABClonal) was used and chemiluminescent signal was captured using an Amersham™ Imager 600 with a 60-second exposure time.

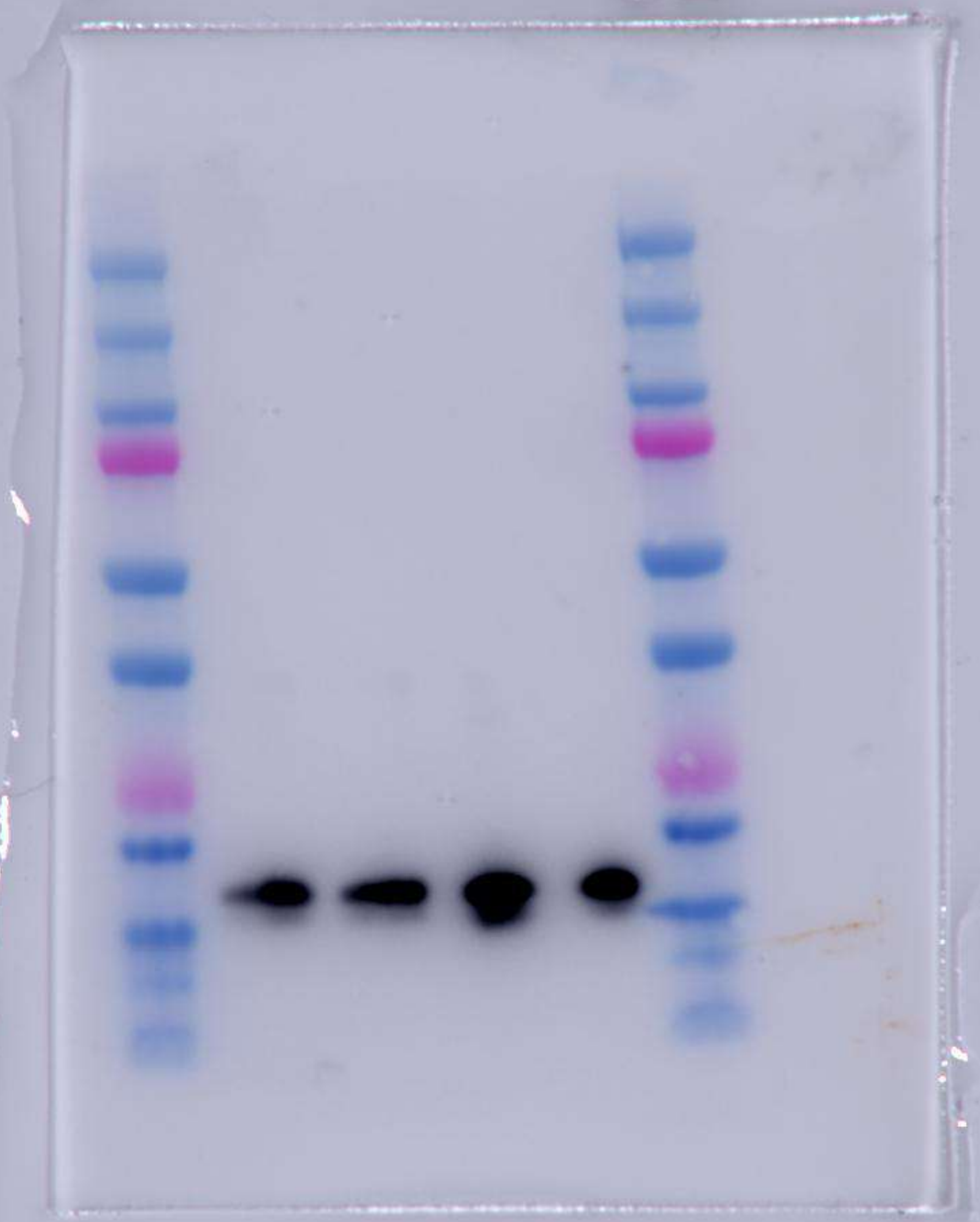

Western blot of Histone 3 K9 trimethyl levels measured in histone extracts with the primary antibody: anti-Histone H3K9me3 (A-4036, Epigentek). Lane 2 and 3 are Mithramycin A-treated samples. Lane 4 and 5 are untreated rotifers cultures. Lanes 1 and 6 are the Precision Plus Protein™ Standard used as molecular weight marker.

HRP-conjugated Goat anti-Rabbit IgG (H+L) secondary antibody (#AS014, ABClonal) was used and chemiluminescent signal was captured using an Amersham™ Imager 600 with a 60-second exposure time

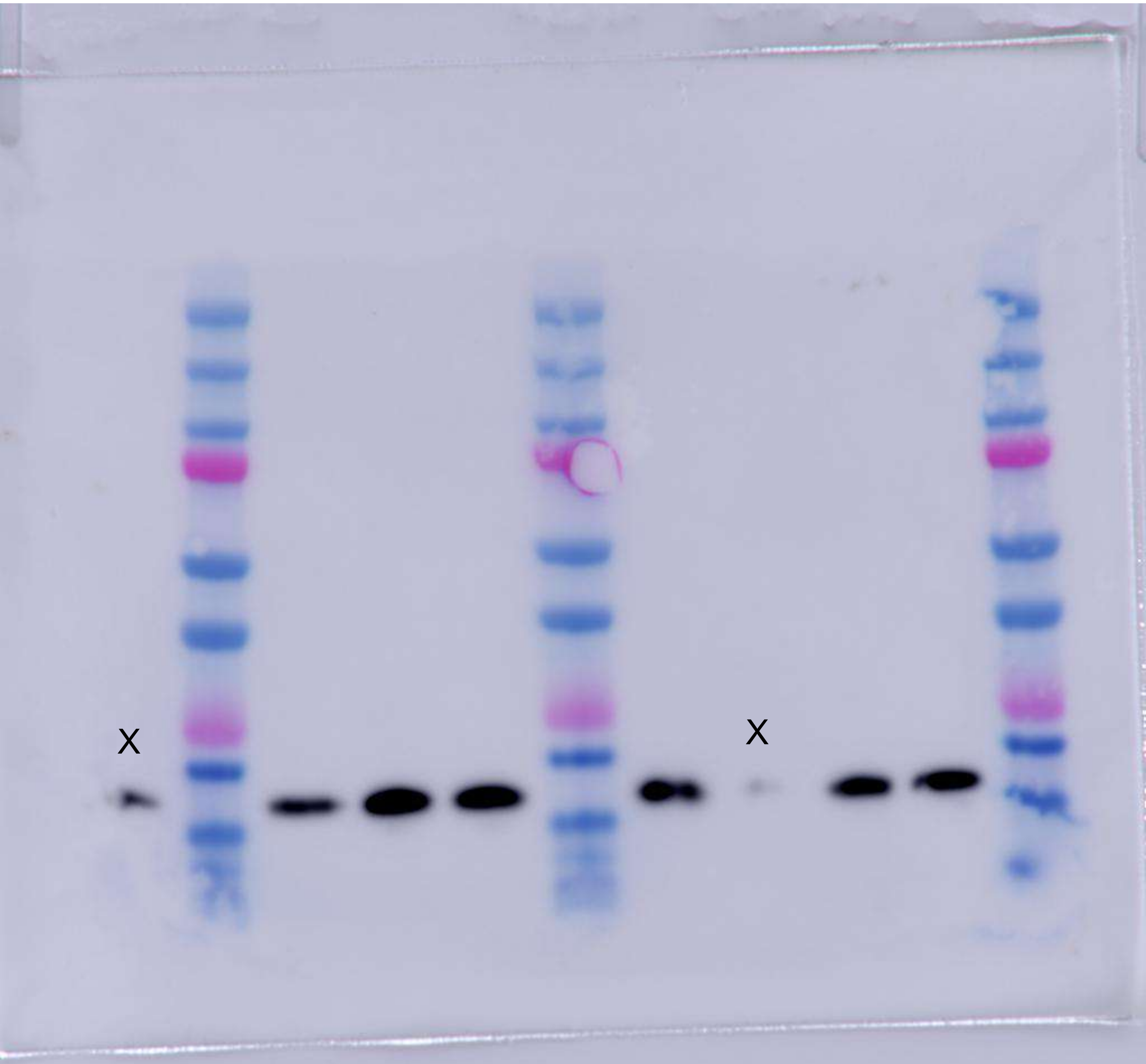

Western blot of Histone 3 levels measured in histone extracts with the primary antibody: anti-Histone H3 (ab24834, Abcam). Lane 3, 4 and 5 are β-hydroxybutyrate-treated samples. Lane 7, 9 and 10 are untreated rotifers cultures. Lanes 2, 6 and 11 are the Precision Plus Protein™ Standard used as molecular weight marker.

HRP-conjugated Goat anti-Mouse IgG (H+L) (#AS003, ABClonal) was used and chemiluminescent signal was captured using an Amersham™ Imager 600 with a 60-second exposure time

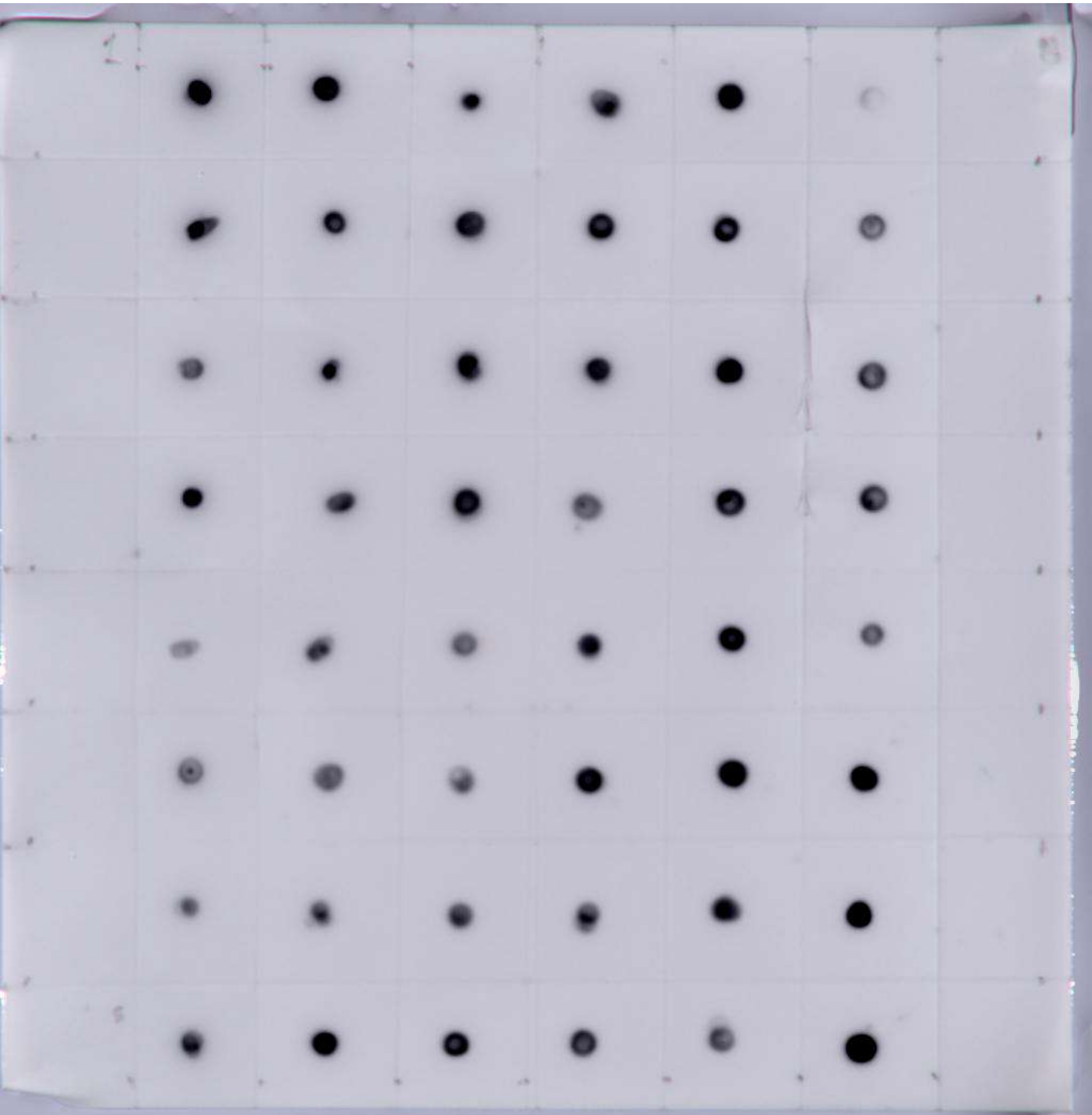

Dot blot of Histone 3 levels measured in all treated and untreated histone extracts with the primary antibody: anti-Histone H3 (ab24834, Abcam)

The samples are displayed in a grid in a separate image: Ctrl water refers to control samples in water; Ctrl DMSO to control samples in DMSO; P is the positive control (HeLa cells treated with sodium butyrate); N is the negative control (an unmodified Histone H3 peptide); SB indicates sodium butyrate-treated samples; BHB refers to β-hydroxybutyrate-treated samples; and Mit denotes samples treated with mithramycin.

HRP-conjugated Goat anti-Mouse IgG (H+L) (#AS003, ABClonal) was used and chemiluminescent signal was captured using an Amersham™ Imager 600 with a 5 minute exposure time

Mit 2.2	P 2	Ctrl water 2.1	Mit 3.1	Ctrl water 1.3	SB 3.3
Ctrl DMSO 3.2	Mit 3.2	BHB 1.3	Mit 1.2	Ctrl water 2.2	Ctrl DMSO 1.1
Ctrl water 3.1	BHB 2.3	Ctrl DMSO 2.1	Ctrl water 3.3	Mit 2.3	Mit 1.3
SB 1.1	Ctrl water 1.1	BHB 1.1	BHB 3.3	Ctrl DMSO 2.2	Ctrl water 3.2
P 1	BHB 2.2	SB 2.1	BHB 3.2	Ctrl DMSO 3.1	Ctrl water 2.3
SB 1.3	BHB 1.2	Ctrl water 1.2	SB 3.2	BHB 2.1	Ctrl DMSO 3.3
SB 2.2	Ctrl DMSO 1.2	Mit 1.1	BHB 3.1	Mit 3.3	SB 2.3
Mit 2.1	SB 3.1	Ctrl DMSO 2.3	SB 1.2	Ctrl DMSO 1.3	P 3

Grid showing the arrangement of all treated and untreated histone extracts samples used in the dot blot, probed with an anti-Histone H3 antibody. Ctrl water refers to control samples in water; Ctrl DMSO to control samples in DMSO; P is the positive control (HeLa cells treated with sodium butyrate); N is the negative control (an unmodified Histone H3 peptide); SB indicates sodium butyrate-treated samples; BHB refers to β-hydroxybutyrate-treated samples; and Mit denotes samples treated with mithramycin.

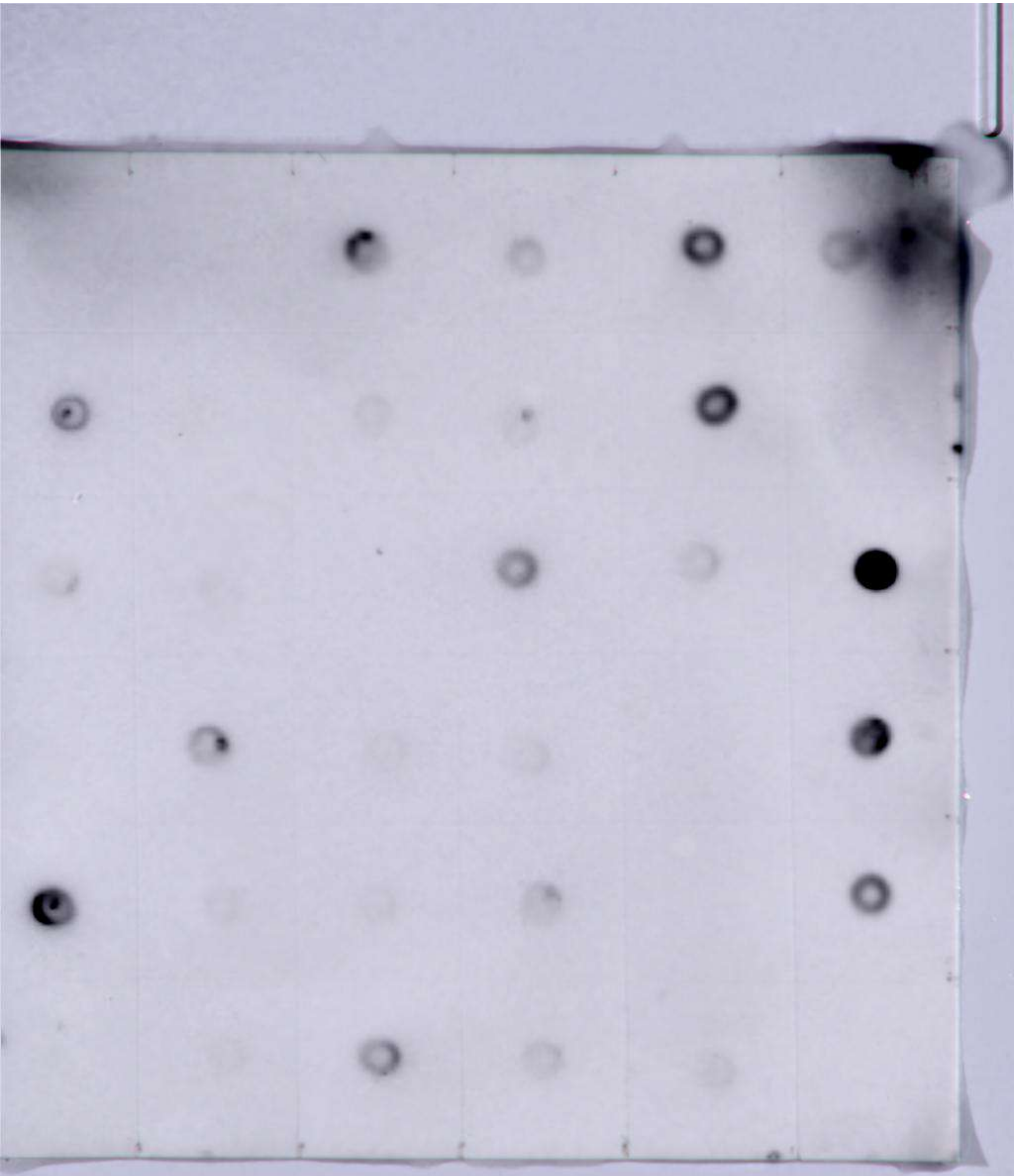

Dot blot of Control (water) and β-hydroxybutyrate-treated samples probed with anti-histone H3 pan-acetyl antibody: anti-Histone H3 (acetyl K4+K9+K14+K18+K23+K27) ab300641, Abcam.

The samples are displayed in a grid in a separate image: Ctrl water refers to control samples in water; Ctrl DMSO to control samples in DMSO; P is the positive control (HeLa cells treated with sodium butyrate); N is the negative control (an unmodified Histone H3 peptide) and BHB refers to β-hydroxybutyrate-treated samples.

HRP-conjugated Goat anti-Rabbit IgG (H+L) secondary antibody (#AS014, ABClonal)was used and chemiluminescent signal was captured using an Amersham™ Imager 600 with a 5 minute exposure time

Blank	*N 3*	BHB 3.1	Ctrl water 3.2	BHB 2.2	BHB 2.1
P 2	Blank	Ctrl water 2.3	Ctrl water 2.2	BHB 1.3	Blank
Ctrl water 3.1	Ctrl water 3.1	*N 2*	BHB 2.3	Ctrl water 3.3	P 3
Blank	BHB 1.1	Ctrl water 1.2	Ctrl water 1.1	Blank	BHB 3.3
P 1	Ctrl water 1.3	Ctrl water 1.2	Ctrl water 1.1	*N 1*	BHB 1.2
Blank	Ctrl water 1.3	BHB 3.2	Ctrl water 3.2	Ctrl water 2.1	Blank

Grid showing the arrangement of control water (Ctrl water) and β-hydroxybutyrate-treated samples (BHB) used in the dot blot, probed with an anti- anti-Histone H3 (acetyl K4+K9+K14+K18+K23+K27) antibody.

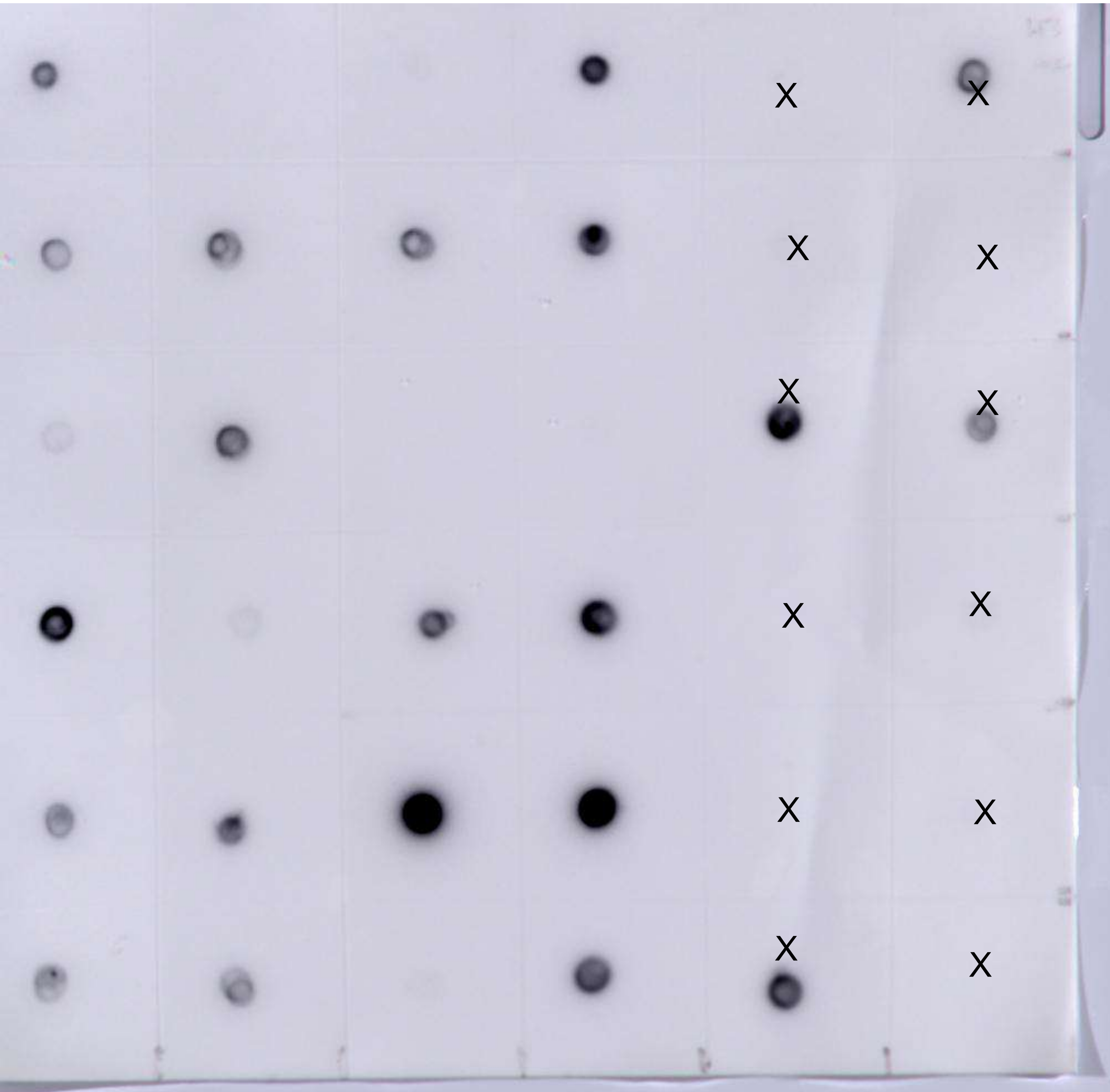

Dot blot of Control (water) and sodium butyrate-treated samples probed with anti-histone H3 pan-acetyl antibody: anti-Histone H3 (acetyl K4+K9+K14+K18+K23+K27) ab300641, Abcam.

The samples are displayed in a grid in a separate image: Ctrl water refers to control samples in water; Ctrl DMSO to control samples in DMSO; P is the positive control (HeLa cells treated with sodium butyrate); N is the negative control (an unmodified Histone H3 peptide) and SB indicates sodium butyrate-treated samples.

HRP-conjugated Goat anti-Rabbit IgG (H+L) secondary antibody (#AS014, ABClonal)was used and chemiluminescent signal was captured using an Amersham™ Imager 600 with a 10 minute exposure time

SB 1.3	N1	Ctrl water 3.2	SB 1.2
Ctrl water 2.2	SB 3.1	Ctrl water 1.1	SB 2.2
Ctrl water 1.2	SB 2.3	N2	N3
P 1	Ctrl water 1.3	SB 2.1	P 3
Ctrl water 2.1	SB 3.3	P 2	SB 3.2
Ctrl water 3.3	Ctrl water 2.3	Ctrl water 3.1	SB 1.3

Grid showing the arrangement of control water (Ctrl water) and sodium butyrate-treated samples (SB) used in the dot blot, probed with an anti- anti-Histone H3 (acetyl K4+K9+K14+K18+K23+K27) antibody.

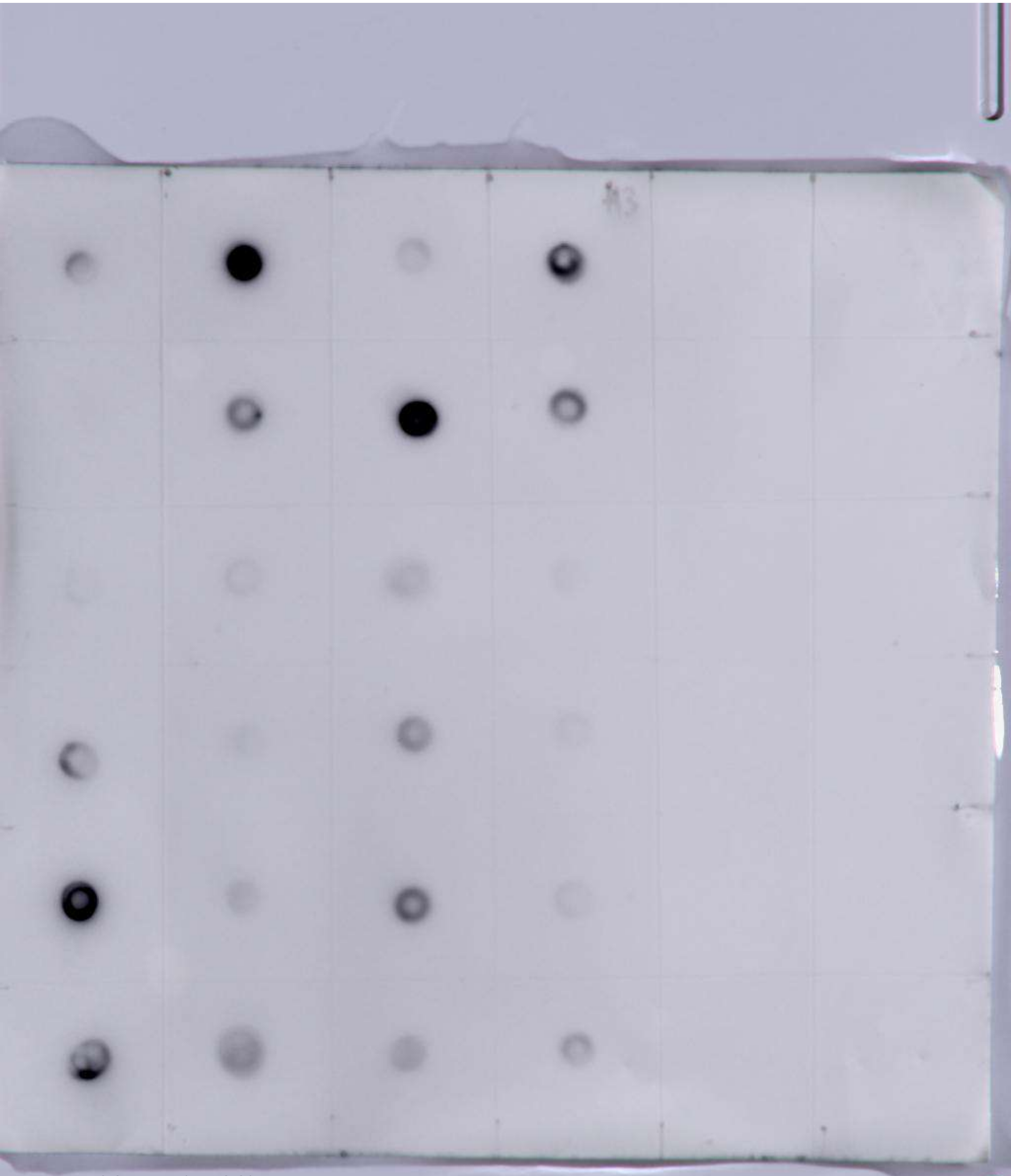

Dot blot of Control (DMSO) and mithramycin A-treated samples probed with anti-H3K9me3 antibody: anti-Histone H3K9me3 (A-4036, Epigentek).

The samples are displayed in a grid in a separate image: Ctrl water refers to control samples in water; Ctrl DMSO to control samples in DMSO; P is the positive control (HeLa cells treated with sodium butyrate); N is the negative control (an unmodified Histone H3 peptide) and Mit denotes samples treated with mithramycin.

HRP-conjugated Goat anti-Rabbit IgG (H+L) secondary antibody (#AS014, ABClonal)was used and chemiluminescent signal was captured using an Amersham™ Imager 600 with a 10 minute exposure time

Mit2.2	P 2	Mit2.3	Ctrl DMSO 2.1
Blank	Ctrl DMSO 3.1	P 3	Ctrl DMSO 2.3
Mit3.1	Mit1.2	Ctrl DMSO 3.2	Mit1.1
Ctrl DMSO 3.3	Mit2.1	Ctrl DMSO 3.2	Mit3.2
P 1	Ctrl DMSO 2.2	Ctrl DMSO 1.1	Mit1.3
Ctrl DMSO 1.3	Ctrl DMSO 2.2	Ctrl DMSO 1.2	Mit 3.3

Grid showing the arrangement of control DMSO (Ctrl DMSO) and mithramycin A-treated samples(Mit) used in the dot blot, probed with an anti-H3K9me3 antibody.
